# Earth-abundant 3d transition metals on the rise in catalysis

**DOI:** 10.3762/bjoc.18.8

**Published:** 2022-01-07

**Authors:** Nikolaos Kaplaneris, Lutz Ackermann

**Affiliations:** 1 Institut für Organische und Biomolekulare Chemie, Georg-August-Universität Göttingen, Tammannstraße 2, 37077 Göttingen, Germany; 2 Wöhler Research Institute for Sustainable Chemistry (WISCh), Georg-August-Universität Göttingen, Tammannstraße 2, 37077 Göttingen, Germany

**Keywords:** C–H activation, 3d transition metals, green chemistry, late-stage functionalization, sustainability

Transition metal catalysis has emerged as a transformative platform for the assembly of increasingly complex compounds, with enabling applications to natural product syntheses, crop protection or medicinal chemistry. Particularly, cross-coupling reactions [1], as well as alkene and alkyne metathesis [2–3], have considerably changed the art of molecular synthesis, with a major impact on neighboring disciplines, such as molecular biology or materials sciences. Despite of these indisputable advances, this approach has, thus far, predominantly relied on precious, often toxic, 4d and 5d transition metals, most prominently palladium, rhodium and iridium. In sharp contrast, the use of less expensive and less toxic Earth-abundant 3d transition metals continues to be underdeveloped. This lack of viable catalysis strategies involving 3d transition metals is largely due to a limited knowledge on the working mode of these metal catalysts, which often involve single-electron-transfer-based redox events. As a consequence, there is a strong demand for efficient and reliable transformations to form C–C and C–heteroatom bonds, thereby providing a more sustainable future for, among others, drug development in generations to come. Particularly, Nobel prize-winning palladium-catalyzed cross-coupling reactions have been recognized by the practitioners in agrochemical and pharmaceutical industries as one of the most powerful methods for molecular assembly. With regard to the cost of goods and the allowance of trace metal impurities in medicinally relevant compounds, 3d transition metal complexes, such as those of iron, copper, cobalt or nickel, represent exciting, more sustainable alternatives. Furthermore, metal-catalyzed cross-couplings do require prefunctionalizations on both substrates and generate stoichiometric quantities of undesired chemical waste, thus reducing the sustainability of these catalytic transformations. To address these major limitations, the past decades have witnessed major momentum in metal-catalyzed C–H activation [4–5] as a more resource-economical strategy. This approach involves the efficient and selective cleavage of otherwise inert, yet omnipresent C–H bonds. This strategy avoids a variety of steps and reduces the amount of chemical waste. Very recently, notable advances have been accomplished with environmentally benign, Earth-abundant 3d transition metals [6–7]. The articles in this thematic issue dedicated to advances in Earth-abundant 3d metal catalysis highlight the unique power of 3d transition metals with a topical focus on homogeneous catalysis. Applications of this strategy range from late-stage functionalization to modern photocatalysis and electrocatalysis, with contributions from around the globe, including Brazil, China, Japan, Germany, India, and South Korea, among others.

The increasing use of C–H activations in academic and industrial laboratories calls for a critical analysis of these methods to enable an efficient transition of these methods. Hence, manganese-catalyzed C–H functionalization for late-stage functionalizations of biomolecules and drug-like scaffolds are summarized [8]. Likewise, 3d transition metal-catalyzed C–H functionalization enabled the de novo assembly of bioactive molecules [9]. The full potential of the mild nature of C–H functionalization is unlocked by the merger with modern photochemistry and electrocatalysis manifolds. In this context, recent advances were realized by the combination of photoredox catalysis and nickel-catalyzed C–H functionalization [10]. Iron complexes are typically cost-effective and nontoxic, and therefore, their use in domino processes represents an outstanding prospect for sustainable organic syntheses [11]. Directed C–H activations have been developed as increasingly amenable tools for proximity-induced C–H functionalizations. In this thematic issue, strategies are presented that guarantee position-selectivity in copper-mediated isoindolin-1-one synthesis [12] as well as in copper-catalyzed aminations of ferrocenes [13]. The exploitation of the innate reactivity of organic molecules can allow for indirected C–H transformations and herein, homolytic C–H cleavages are described for transformative manganese-catalyzed brominations of tertiary C–H bonds [14]. Finally, electrooxidation enabled the site-selective alkynylation of tetrahydroisoquinolines within a TEMPO/copper regime [15].

As the editor of this issue on Earth-abundant 3d metal catalysis, it was a wonderful experience to experience the diversity of 3d transition metal catalysis, which continues to address key challenges of sustainable modern molecular syntheses. The senior author owe a great debt of gratitude to all of the authors for their dedication and time in contributing to this effort. Finally, the senior author thank the staff at the *Beilstein Journal of Organic Chemistry* for their assistance.

Nikolaos Kaplaneris and Lutz Ackermann

Göttingen, December 2021
